# Children and adolescents coping with home isolation and social distancing during Covid-19 in Qatar: a cross sectional study with qualitative items

**DOI:** 10.1186/s40359-023-01183-6

**Published:** 2023-05-06

**Authors:** Abduljaleel Abdullatif Zainel, Suhad Daher-Nashif, Alyaa Nasser Al-Maadeed, Hamda Abdulla Qotba, Hanan Al Mujalli, Sadriya Mohd Al-Kohji

**Affiliations:** 1 Primary Health Care Corporation, Doha, Qatar; 2 School of Medicine, Keele University, Keele, UK; 3 Doha Institute for Graduate Studies, Doha, Qatar

**Keywords:** COVID-19 pandemic, Children, Adolescents, Psychosocial implications, Coping strategies, Qatar

## Abstract

**Background:**

Covid-19 related studies report psychological impacts during home isolation and social distancing. Despite that, children and adolescents were able to adopt coping strategies that assisted in lowering severe levels of psychological disorders. This study aims to report on the psychosocial implications of social distancing and isolation on children of different nationalities who reside in Qatar, and to reveal their coping ways.

**Methods:**

This is a cross sectional study with qualitative component at its end. The study is a part of a larger study that reported the results of a national screening for psychological disorders experienced by children and adolescents in Qatar. A bilingual online questionnaire included close-ended and one open-ended question to screen for psychological changes and identify coping strategies practiced by children and adolescents (7–18 years) during home-isolation and social distancing. The quantitative questionnaire had five main sections as follows: the sociodemographic characteristics, Spence Children’s Anxiety Scale, Kutcher Adolescent Depression Scale, and Clinical Anger Scale). The last section screened for eight different coping strategies. The summative content analysis was used to analyze the open-ended question “What practices do you do at home that make you happy?”. First, open coding was used (for identification), followed by the axial coding (for comparison), and lasted by sorting of coping strategies inductively.

**Results:**

Six thousand six hundred and eight (6608) subjects participated between June 23 and July 18, 2020. The clinical outcomes of the study had varying prevalence and levels of severity, which ranged from mild to severe. Higher prevalence was noted for adjustment disorder 66.5% (n = 4396), and generalized anxiety 60% (n = 3858), in comparison to depression 40% (n = 2588). Additionally, participants reported using cognitive, spiritual, social, and physical coping strategies. Eight higher order themes were identified to reflect the coping strategies: playing with siblings or pets, gardening, cooking, practicing arts and crafts, and doing chores. Furthermore, Sociodemographic factors such as ethnicity, religion and family status played a considerable role in choosing the type of coping strategy.

**Conclusion:**

The uniqueness of the study is bringing the psychosocial implications of social distancing through the voices of children and adolescents, and coping strategies from their perspective. These results are of importance for educational and healthcare systems that are recommended to collaborate even in “normal” times to prepare these age categories for any future crises. The importance of daily lifestyle and family is highlighted as protectors, and crucial factors in emotional management.

## Introduction

Reports from different studies and health organizations showed lower numbers of infections and deaths from Corona virus among children and adolescents, compared to adults and older adults. Nevertheless, studies revealed severe psychosocial implications that impacted them in different ways [1–3]. Due to being quarantined and socially isolated from their peers and the society, as it was associated with mandated school closures and lack of outdoor activities. That being said, those drastic changes have led to increased anxiety, distress, anger, adjustment disorder, and regression in academic performance and more [1–5].

In a comprehensive review of 77 articles between 2020 and 2022, de Miranda et al. (2020) reported that high rates of anxiety, depression, and post-traumatic symptoms were identified among children due to the pandemic [6]. A meta-analysis of twenty-three studies (21 cross-sectional studies and 2 longitudinal studies) from China and Turkey with 57,927 children and adolescents, showed that the pooled prevalence of depression, anxiety, sleep disorders, and posttraumatic stress symptoms were 29%, 26%, 44% and 48% respectively [7]. They also reported that the subgroup meta-analysis revealed that adolescents and females exhibited higher prevalence of depression and anxiety compared to children and males, respectively.

In comparison, very few studies reported the impacts of the pandemic on children and adolescents in Arab Nations. Among those, the study of Zainel et al. (2021) that included a screening of 6608 children and adolescents age ranging from 7 to 18 years, 66.5% screened as mild levels of adjustment disorder, almost 40% screened as mild and moderate levels of depression, and almost 50% screened as mild levels of general anxiety. Sayed et al. (2021) study in Saudi Arabia found that among 537 participants symptoms of no, minimal, mild and potential PTSD were identified in 15.5%, 44.1%, 27.4% and 13.0% of children/adolescents, respectively. The two studies as they report mild to moderate levels of psychological disorders, draw more attention on what factors supported children and adolescents to cope with the drastic daily changes [5, 8].

There are many lessons we can learn from children and adolescents’ experiences during Covid-19 pandemic, especially when home-isolation and social distancing are forced stressors, and coping begins as a spontaneous response to these stressors and continues to be part of a routine. Literature defines coping as a combination of a behavior and cognitive effort to reduce distress [9–11]. While there is not one way to categorize coping strategies, the most known taxonomies are: adaptive and maladaptive, problem-focused and emotion-focused, social support and cognitive reframing in addition to spiritual coping [10, 12]. Coping categorization is usually constructed using exploratory factor analysis, rationale sorting, or confirmatory factor analysis [12], where strategies are grouped into the aforementioned coping taxonomies depending on how the behavior responds to the stressor.

This article aims to shed light on the voices of children and adolescents and learn from their practices and life-style changes that led them to cope with home-isolation and social distancing. This article presents part of larger study entitled “Psychological and coping strategies related to home isolation and social distancing in children and adolescents during the COVID-19 pandemic: Cross-sectional study”, in which children and adolescents in Qatar reported practicing different coping strategies such as praying (64% often & all the time), asking for support from others (62% often & all the time), choosing to receive information from official channels (79% often & all the time), spending more time with family (79% often & all the time) [5]. In this article we highlight the qualitative component of the study through which we bring participants’ narratives on their coping strategies. Furthermore, as supportive background part of the quantitative findings that are relevant to the qualitative component, and give strong background to our claims all along the article. Such data can help parents and healthcare providers understand what helped children and adolescents cope with a global pandemic while forcefully being at home and distanced from the society, and will help in planning tailored future plans for crises such as in pandemic times. This is the first study of its type in Qatar, and among the very few studies on children and adolescents in the Arab region. The importance of the study emerges from the unique context of the country. Qatar is considered as highly culturally diverse, when most of its 2.9 million population are of migrant workers with almost 100 nationalities, and tens of cultural and religious backgrounds. This diversity is reflected on the daily lives of families and the ways they and their children cope with crises such as during the COVID-19 pandemic. Hence, this study will inform stakeholders through children and adolescents’ voices, on culturally competent plans to prepare for children and adolescents for future crises. The study was managed by the clinical research department at PHCC-Qatar.

## Method

The study has been performed in accordance with the Declaration of Helsinki and have been approved by the Primary Health Care Corporation’s Institutional Review Board (PHCC-IRB) in Qatar.

### Study design

This is a quantitative crossed sectional study with qualitative component. An online questionnaire was introduced to the PHCC’s Health Information Management (HIM) department who produced a mobile text with a direct link to an online questionnaire, the text was received by parents registered at PHCC who have 7–12-year-old children and/or 13–18-year-old adolescents. Two versions were provided (one in Arabic and one in English) alongside with an explanatory introduction of the study and an informed consent form. Prior to enrollment of any child in this study, his/her parent was requested to sign an informed consent form on behalf of the participant.

### Inclusion and exclusion criteria

The inclusion criteria were children and adolescents aged 7–18 years old, registered within PHCC database and were living in Qatar during COVID 19 pandemic. We excluded any children below the age of 7 years old as well as children and adolescents with intellectual disadvantages, being unable to communicate their thoughts.

Participants with intellectual disabilities were excluded from the study since the aim of the study was to screen for psychological changes and identify coping strategies practiced by children and adolescents for the duration of home-isolation and not in a setting controlled by a facilitator.

### Study instruments

The quantitative questionnaire had five main sections as follows: the sociodemographic characteristics section was answered by parents, three sections included psychological screening questions which combined (Spence Children’s Anxiety Scale [SCAS], Kutcher Adolescent Depression Scale [KADS], Clinical Anger Scale [CAS]) tailored to fit the questionnaire and home-isolation setting. The last section screened for eight different coping strategies: (1) practicing prayers and worship, 2) asking for support from people who care and understand own feelings, (3) choosing to receive accurate information from official sources about the pandemic, (4) limiting the use of electronic devices, (5) spending more time with family members, (6) participating in at-home activities such as cooking or playing board games, (7) exercising, and (8) arranging sleep time. These practices were found by experts in the PHCC research department, PHCC community engagement group and community medicine department, as well as experts in social sciences and social work studies from Doha Institute for Graduate Studies. According to Aldwin (2004), “culture can affect the way stressors are reacted to, and generally affect the choice of coping strategies” (p. 565) [13]. Thus, the above mentioned eight main practices were found to be applicable to the lifestyle of children and adolescents in Qatar and were included in the quantitative part.

Since “there is not a specific strategy for identifying coping sub-categories that can be reliability reported” [12] (p. 217), The aforementioned strategies were grouped into four main categories using a rationale classification for constructing lower order coping categories. Rationale sorting involves “grouping coping strategies that share similar features, and it is supported by conceptual clarity” [12] (p. 222) The four coping categories can help with understanding how those practices hindered or aided scoring higher in symptoms related to the addressed psychological disorders in this study, while taking into consideration the differences between each disorder. The four main coping categories are: spiritual/emotional, physical, cognitive, and social.^1^

In this article we bring focus to the qualitative component of the study where we used an open-ended question regarding coping strategies used by children and adolescents at home. The question was “What practices do you do at home that make you happy?”. Although this method generates less data than interviews, it was one appropriate approach utilized during the pandemic by many studies, to generate data across large samples [14, 15]. Hence, we will present parts of the study that document these strategies by answering the following question. Although parents were directed to assist children and adolescents when filling out the answers if needed, as mentioned above, parents were only asked to fill out the first section answering sociodemographic characteristics.

### Data analysis

For the quantitative data analysis, frequencies with percentages were calculated for categorical variables, and mean (SD) values were calculated for discrete variables. Pearson correlation coefficients were calculated to assess the correlation among the final psychological diagnoses (anger, adjustment disorder, depression, general anxiety, and separation anxiety) and the 4 categories of coping strategies (spiritual/emotional, social, physical, and cognitive). A *P* value of 0.05 (two-tailed) was considered to indicate statistical significance. SPSS (version 23.0, IBM Corp) was used for the statistical analysis. The scoring method was linked to the scoring system of the tools mentioned above and comparable to the severity levels of each diagnosis in the DSM-5 (mild, intermediate, or severe). The final scoring system of the questionnaire was standardized across all screening questions to ensure quality of screening and participants’ ability to answer them.

As for the qualitative data, it indicated narratives written in first-person, that is by children or adolescents. We used summative content analysis to analyze the answers. A summative content analysis is an inductive analysis that involves counting and comparisons, usually of keywords or content, followed by the interpretation of the underlying context [16]. We began the analysis with open coding (for identification), followed by the axial coding (for comparison), then rationale sorting of coping strategies using a bottom-up approach, we identified higher orders and then grouped several strategies in lower orders. Skinner et al. (2003) argue that “it is important to identify higher order themes to reflect on distinctions of the lower order coping activities” (p. 225) [12]. The analysis of qualitative data ended with the descriptive themes of analysis that will be presented in the following section.

## Results

### Participants’ characteristics

Six thousand six hundred and eight (6608) subjects participated in the study between June 23 and July 18, 2020. Tables 1 and 2 present the frequency distribution of sociodemographic characteristics, final psychological diagnoses, and distribution of coping categories used by children and adolescents during home isolation in Qatar during March-August 2020 (N = 6608).

**Table 1:** Frequency distribution of sociodemographic characteristics of participants (N = 6608)

Variable	Category	Frequency (N)	Percent (%)
Language	English	2983	45.1
	Arabic	3625	54.9
Age	7 to 12 years	4148	62.8
	13 to 18 years	2460	37.2
Gender	Male	3354	50.8
	Female	3254	49.2
Nationality	Qatar	1374	20.8
	Arab	2237	33.9
	South Asia	1907	28.9
	Others	1090	16.5
Education	Primary	3220	48.7
	Middle	1733	26.2
	Secondary	1355	20.5
	Completed secondary	300	4.5
Income	Up to 5000 QR	627	9.5
	5001–10,000 QR	1443	21.8
	10,001–20,000 QR	2034	30.8
	20,001–40,000 QR	1448	21.9
	40,001–60,000 QR	598	9
	> = 60,001 QR	458	6.9

**Table 2:** Psychological disorders & coping strategies used among the participants (N = 6608)

Variable	Category	Frequency (N)	Percent (%)
Anger	No anger	4784	72.4
	Mild anger	1740	26.3
	Severe anger	84	1.3
Adjustment disorder	No adjustment disorder	1349	20.4
	Mild adjustment disorder	4396	66.5
	Moderate to severe disorder	863	13.1
Depression	No depression	3760	56.9
	Mild depression	1927	29.2
	Moderate depression	661	10
	Severe depression	260	3.9
General anxiety	No anxiety	2646	40
	Mild anxiety	3285	49.7
	Intermediate anxiety	573	8.7
	Severe anxiety	104	1.6
Separation Anxiety	No Separation Anxiety	5077	76.8
	Possible Separation Anxiety	1531	23.2
Spiritual/emotional coping strategy	Did not use spiritual/emotional coping strategy	508	7.7
	Somewhat felt comfortable and maintained practicing spiritual/emotional coping strategy	2439	36.9
	Always felt comfortable and maintained practicing spiritual/emotional coping strategy	3661	55.4
Cognitive coping strategy	Did not use cognitive coping strategy	701	10.6
	Somewhat felt comfortable and maintained practicing cognitive coping strategy	4220	63.9
	Always felt comfortable and maintained practicing cognitive coping strategy	1687	25.5
Physical coping strategy	Did not use physical coping strategy	1931	29.2
	somewhat felt comfortable and maintained practicing physical coping strategy	3539	53.6
	Always felt comfortable and maintained practicing physical coping strategy	1138	17.2
Social coping strategy	Did not use social coping strategy	379	5.7
	Somewhat felt comfortable and maintained practicing social coping strategy	2260	34.2
	Always felt comfortable and maintained practicing social coping strategy	3969	60.1

As shown in Table 1 the distribution of sociodemographic characteristics, children made up two-thirds of the sample, while adolescents comprised approximately one-third of it. Approximately half of the participants were male, and the other half were female. Nearly half of the participants were in primary school and about three-quarters came from middle-income families. Table 2 reports final psychological disorders screened, and coping strategies adopted by children and adolescents. Most psychological effects were mild to moderate, and the coping strategies included spiritual/emotional, cognitive, social, and physical.

### Symptoms and age-related coping strategies

The results show higher rates of practicing spiritual strategies such as spending time with the family 51.9%; asking for information from parents and official channels (49.3%), asking for support from parents (36.2%), and prayer (33.8%) (Table 3).

**Table 3:** Frequency distribution of symptoms related to each coping strategy used by participants

The main variables	The sub-categorical variables	None of the time N (%)	Rarely N (%)	Some of the time N (%)	Often N (%)	All the time N (%)
Spiritual/emotional coping strategy	Practicing prayer and religious worship more	374 (5.7)	541 (8.2)	1460 (22.1)	2001 (30.3)	2232 (33.8)
	Asking for support from people who care about me and understand his/her feelings, such as his/her parents, schools’ surroundings	429 (6.5)	667 (10.1)	1404 (21.2)	1713 (25.9)	2395 (36.2)
Cognitive coping strategy	Choosing to receive accurate information from parents and official channels instead of rumors from peers	165 (2.5)	304 (4.6)	916 (13.9)	1965 (29.7)	3258 (49.3)
	Limiting using of electronic devices	1825 (27.6)	2221 (33.6)	1838 (27.8)	516 (7.8)	208 (3.1)
Social coping strategy	Spending more time with the family	89 (1.3)	330 (5.0)	958 (14.5)	1803 (27.3)	3428 (51.9)
	Doing in-home activities with the family, such as cooking a meal or playing board games	326 (4.9)	857 (13.0)	2254 (34.1)	1939 (29.3)	1232 (18.6)
Physical coping strategy	Doing more exercise	896 (13.6)	1947 (29.5)	2280 (34.5)	1006 (15.2)	479 (7.2)
	Arranging of sleep time	1160 (17.6)	1460 (22.1)	1805 (27.3)	1337 (20.2)	846 (12.8)

Since the study population is very diverse, we found differences in the relationship between different age categories and coping strategies (Table 4). The differences across age groups were moderate in most cases.

**Table 4:** Association between age categories of the study’s population to the coping strategies adopted by them

Variable	Category	7–12 years old	13–18 years old	7–18 years old	*P* value
Spiritual/emotional coping strategy	Did not use	259 (6.2%)	249 (10.1%)	508 (7.7%)	< .001
	Somewhat felt comfortable and maintained practicing	1409 (34.0%)	1030 (41.9%)	2439 (36.9%)	
	Always felt comfortable and maintained practicing	2480 (59.8%)	1181 (48.0%)	3661 (55.4%)	
Cognitive coping strategy	Did not use	360 (8.7%)	341 (13.9%)	701 (6.6%)	< .001
	Somewhat felt comfortable and maintained practicing	2590 (62.4%)	1630 (66.3%)	4220 (63.9%)	
	Always felt comfortable and maintained practicing	1198 (28.9%)	489 (19.9%)	1687 (25.5%)	
Physical coping strategy	Did not use	1148 (27.7%)	783 (31.8%)	1931 (29.2%)	.001
	Somewhat felt comfortable and maintained practicing	2264 (54.6%)	1275 (51.8%)	3539 (53.6%)	
	Always felt comfortable and maintained practicing	736 (17.7%)	402 (16.3%)	1138 (17.2%)	
Social coping strategy	Did not use	160 (3.9%)	219 (8.9%)	379 (5.7%)	< .001
	Somewhat felt comfortable and maintained practicing	1265 (30.5%)	995 (40.4%)	2260 (34.2%)	
	Always felt comfortable and maintained practicing	2723 (65.6%)	1246 (50.7%)	3969 (60.1%)	

### What did make the children and adolescents happy?

In the qualitative open-ended question, participants presented seven types of strategies they practiced in-home to entertain themselves and overcome challenge of the lockdown and social distancing. Seventeen different strategies were identified in participants’ answers to the question “What practices do you do at home that make you happy?”. The 17 strategies are considered lower order coping practices, the ones that shared similar attributions such as (what environment can they be performed, and if it takes others to participate) were grouped (Table 5), the outcome is 8 distinct higher order coping strategies.

**Table 5:** Higher and Lower Order Coping Strategies Practiced by Children & Adolescents in Qatar During Home-Isolation & Social Distancing

Higher order	Lower order
Playing	Playing with siblings or parents
	Playing with pets
Practicing activities	Gardening
	Cooking
	Arts and crafts
Contributing to chores	Helping parents or grandparents
	Helping younger siblings
Using technology	Online and video games
	Socializing virtually with family and friends
	Joining online classes and workshops
Watching movies & listening to music	Watching movies and listening to music or playing an instrument
Reading books	Reading books
Religious practices	Praying
	Reading holy book
	Reciting dua
Exercising	Indoor exercising
	Outdoor exercising

### Playing

“We change the organization of home furniture and make a tent in different places at home”,

“Cooking, exercising, sitting with my family, talking about family affairs, playing with my brothers and sisters, young and old, talking to my grandmother and playing with her”.

The above two quotations like many others, indicate playing as a coping strategy which hindered experiencing psychological symptoms related to depression, anxiety, and anger, and assisted with boosting participants moods.

#### With siblings or parents

Participants of the study also highlighted playing with family members during lockdown. A participant from East Timor for example said “dancing, helping and playing with my family”. A Syrian child answered:

“Drawing and playing with colors and playing with my little brothers and we spend time mostly between TV and iPad and preparing food and arranging the house and going out for walks with my family in Aspire Park”.

Another participant mentioned:

“I chat with my friends as I am not able to go out. I play board games with my parents. I do craft activities with clay dough and paper cuttings and cardboards and play with my available toys without asking to buy new until the end of this situation which I have promised my mom. I love playing with dad whenever at home. I feel very excited to play with my dad and speaking to him about everything. I am more organized which makes me happier as my mom appreciates and tells everyone about which makes me happier”.

Few respondents mentioned playing outdoor with their siblings, especially those who had back yards and spaces. For example, a Qatari child answered, “I play football at my house with my brothers and riding a bike”. Many participants (n-145) mentioned playing with somebody or engaging in interactive playing, indicating that socializing with someone while playing is a key factor in coping with depressive symptoms.

#### With pets

Socializing was mentioned with family members, also pets. Playing and taking care of animals and pets was mentioned by 38 participants. For example, a 17-year-old Syrian mentioned.

I found two baby pigeons and a baby sparrow outside, their parents left them so now nearly my whole day is just taking care of them and making sure they continue to live. Other than that, I read fictional books.

Hence, the lockdown was an opportunity to develop caring skills in children and adolescents. The question that should be investigated in future studies, if and how these practices continued or developed after the lock down, and how these practices influenced family relationships and personal development.

### Practicing at-home activities

#### Gardening

Gardening was also mentioned 15 times as an activity. For example, a 16-year-old Indian child answered “Gardening, playing with pets, art”. Although it’s not a game, but it addressed as an enjoyable activity to do with family or alone in the private garden. Another opportunity of helping parents was taking care of the plants inside and outside home. For example, an 8-year-old Indian participant said, “Gardening, feeding birds, playing Ludo, zoom calls with friends and family, cooking with family, watching movies and TV shows, Listening to music”.

#### Cooking

One hundred and Seven participants mentioned cooking as one of the new activities they adopted during the lockdown. For example, a Qatari child answered “Cooking—Playing Football—Drawing—Watching Movies”. Family time could include board games, cards, football, water, and creative games such as coloring, cooking, and painting.

#### Arts & crafts

Arts (fine art) and crafts were mentioned in almost 255 different answers. In some, participants mentioned specific practices like drawing (n = 384), painting (n = 126), coloring (n = 384) and sewing (n = 10).

A 11-year-old Indian said:

“[I] Developed drawing skills, watching TV, playing inside the house, helping for cooking, improving my reading skills, cycling inside the villa compound”

Another 13-year-old Syrian said “Sewing. Crocheted. Handicraft. Learn new skills and languages”.

### Contributing & helping

Being at home with limited ability to entertain children and adolescents can be challenging to parents, however it developed opportunities to be creative while keeping household clean and tidy. Opportunities that would not happen without being forced to spend time together. Many participants reported their contribution to doing house-chores. They talked about it as part of the things they did to cope with the limitations in going outside. For example, one child said “Dancing, reading, helping my younger brother to study, do my household chores, and playing”. Another child answered, “Doing household chores and spending more time with my family members”.

Helping was a word that is used in several answers to express participants contribution and assuring their role and value in the family. As mentioned by a child “By helping mother's routine activity such as cooking, house cleaning, by joining online classes like yoga, music, by doing online workshop like coding, painting”. The kind of support participants revealed to their siblings varied but mostly in their online learning, studying after school and playing with them. For instance, a participant from Pakistan answered the question saying, “Teaching my siblings”. Another Indian child said:

“Helping my mother in home, talking with my brother, talking with my grandparents, Listening to brothers’ online class”.

Some participants also reported new opportunities to re-connect with their grandparents. They reported on spending time with them, sat and talked to them. For example, a Qatari child answered the question by saying:

Help my father and mother, sit down with my grandmother and grandfather, and read some stories in Arabic and English, also, we read *Riad Al-Salihin's* book with my family”.

### Exercising

“I am playing with my siblings all the time, we did swimming at our mini pool at the backyard, singing together & watching movies with my family”.

The quotation above is taken from the answer of 11-year-old Philippian participant, like in his case, many physical activities mentioned by participants. Some were outdoor, and others were indoor.

Outdoor sport activities mentioned by participants included: running, walking, football, basketball, and swimming. Inside physical activities were Yoga, Zumba and Pilates. A child from India said:

“Sometimes I talk to my father and mother and when they don’t have time, I play with my brother, or I watch something in TV. When it is 7pm I go for running and come back to eat food, then maybe I again watch TV or play in the house then we go to sleep”.

Another 7-year-old Brazilian child stated, “Swimming in the pool in my garden and cycle in front of my house”. It is important to mention here that the majority of compounds and residency buildings in Doha includes play spaces, but the time of the lock down coincided with the hot summer days between April and August, making outside activities almost impossible. In addition to being isolated at home, families could not go out to public spaces with their children due the governmental restrictions at the beginning of the first stage of lockdown. Children up the age of 12 were not allowed to be in public close spaces such as malls until the third stage of releasing restrictions in September 2020.

Interesting to note that some participants’ demographic background did not affect their physical activity of choice. I.e., all activities were shared by different participants from different cultures and demographic backgrounds. This finding indicates that being at home with family, in similar context (Qatar) created kind of similarity in terms of what a child or an adolescent can do in his/her leisure time. For example, a child from the Philippines answered, “Zumba with my family and Aunties”.

### Using electronics

#### Video and online gaming

The type of games varied among participants. The most one used was electronic games. For example, an Egyptian child said: “Because of Corona, I've been connected to my electronic devices more, and I know it's a mistake, and I am afraid of getting out of the house because of the Coronavirus”. Electronic games were followed by board games and online games.

#### Socializing virtually with friends or family

Socializing with friends was mainly remote using video telecommunication and via online games. For example, an Indian child answered.

#### Joining classes & workshops

Online school classes mentioned by most participants as an occupation that distracted them from being in lockdown. In addition, children participated in other online activities such as socializing in social media platforms and attending workshops and leisure group gathering. Being at home with no access to public entertainment spaces, led to a major increase in online activity participation. A 16-year-old Qatari participant answered, “Online training courses (debates, new language, self-development), I do skincare intensive daily, play new video games”. Another eleven-year-old Indian answered:

“By helping mother's routine activity such as cooking, house cleaning, by joining online classes like yoga, music, by doing online workshop like coding, painting”.

Learning new language was mentioned by several children (n = 17). For example, an eleven-year-old Sri Lankan child answered:

“The school summer holidays zoom sessions, learning a new language (Spanish), playing the piano, starting a YouTube channel (educational), learning robotics”.

### Movies and music

One hundred and sixty-eight (168) participants mentioned watching movies/films. The type of movie varied between cartoon, animation, and real fiction movies. Many highlighted which genres of movie they watched, for example: comedy, educational, and family movies. Movie-time and family were cosigned by many participants as well.

Music was also mentioned by 85 participants, it was also indicated using several verbs such as: singing (n = 35), playing instrument such as pianos and guitars (n = 35), dancing (n = 27), and listening to music (n = 25). An 8-year-old child from India said:

“I play with my younger sister and alone as well... I dance... I write stories. I draw pictures. I try to make videos of dance or acting. I learnt to prepare tea and omelet. I watch movies. I have a correct study time”.

#### Reading

Most participants in this category aged between 15 and 17 years old, and only two were 11 and 12 years of age. So, it is worth noting that age is a huge factor in this category. Although “book” appeared in 93 responds, 441 participants in this study mentioned reading as one of the activities practiced during lockdown. Some of the genres mentioned were comics, fiction, history, religion, and novels. Some participants even made this activity as a challenge. For example, a 9-year-old Qatari child said, “Reading story to my mom every 2 days and then I will ask her questions about the story”.

#### Spiritual and religious practices

Despite participants reported nationalities, reading Quran, practicing prayers, and reciting daily dua were mentioned by almost 60 participants. An eleven-year-old Pakistani mentioned:

“I helped my mom a lot in several house works like cooking cleaning washing clothes and dishes... We enjoined a lot the quarantine scheduled our time accordingly with my mom so early morning we are playing sports after *fajer*^2^ prayer and reading holy Quran specially *al Baqarah*^3^ then our whole day like mercy from God *Alhamdullah* [Thanks to God]”

Out of those, thirty participants mentioned praying as a coping practice and connected it to a family member. For example, an 8-year-old Iranian child said, “praying and helping my father and grandfather.” Another 12 years old Indian participant answered:

“Eating together with the family, performing five-times prayer with family in jamaa (together), and corona virus issues discussing with the family”.

Being spiritual and praying is considered as a stress reducer, and an emotional balance inducer. A 16-year-old Pakistani adolescent mentioned:

“Worshiping *Allah* [God] and knowing Allah the most and l try my best to be closed to Allah. Sometimes when I used to pray to Allah that feels me relaxed from the crisis ☺”.

## Discussion

This study aimed to understand how home isolation and social distancing during the COVID-19 pandemic influenced psychologically children and adolescents of different nationalities in Qatar, and the ways they coped with these implications. Children and adolescents voices in this study, taught about successful strategies that helped them cope with pandemic-related stressors such as home isolation and social distancing. Many coping strategies lower the chances of higher levels of separation anxiety, anger, adjustment disorder, depression, and general anxiety to mild and moderate levels [5] than in many other cases around the world [14, 17, 18].

Interesting observations emerge from the answers that added to the study results' uniqueness. Although children and adolescents used similar coping strategies (playing at home, practicing recreational activities, contributing to chores, exercising, watching movies or listening to music, utilizing technology for games and socializing, reading books, and practicing spirituality and praying), adolescents (13–18 years old) seemed to have more interest in reading books than children (7–12 years old). This can be explained by the fact that at the age of 7–8 children are still at the beginning of exploring their reading passion or preferences, while later during adolescence reading becomes and easy mission and passion to pursue.

Another unique finding is how the local culture plays a role in the spiritual coping strategy of choice. Our study found that the majority of participants, especially Muslims, maintained religious practices. For Muslims, religion is part of the daily habits and consistent component of their life style. Hence pandemic and home isolation might increase the rhythm and intensity of practicing religious rituals, also as a way to cope with stressors. Examples shared in the qualitative part were praying, reading the Quran, and reciting dua. Interestingly, participants of other religious beliefs did not mention praying, being spiritual, or reading holy books as part of their coping practices. This indicates the great impact culture can make on people’s coping strategy of choice [13]. Qatar has mosques in every neighborhood and a ministry supervising them. The ministry has been keen on community engagement through activating several initiatives, which empowered members, despite their nationality, to be spiritually connected during the pandemic. Thus, the overall culture of the society played a role in what spiritual coping looks like.

Our study reports that few participants maintained physical exercise, and very few reported exercising all the time. It is concluded to be a direct consequence of not being able to exercise outdoors and in team settings since the community in Qatar is highly vested in sports built on teams like football. However, with the lack of outdoor options, the pandemic paved more opportunities for indoor activities [19]. Only several participants provided examples of exercising outdoors like running and swimming. Others provided in-door options such as Yoga, Zumba, and Pilates. It is worth mentioning here that the weather in Qatar is also a factor that shapes outdoor activities. The lockdown began during spring times in March, but continued to the very hot months of the year June–August, a condition the was a barrier to outdoor sports even for those passionate to it.

Although the use of technology for self-development purposes and online gaming is considered an avoidant coping strategy [20], it is imperative to underline the type of stressor the person is avoiding. Given that the pandemic suppressed people from interacting with others physically, thus, the stressor is a lack of social encounters, or worse, being afraid of getting infected through social encounters. Joining online games and virtual activities with others have proved to help people cope while being in lockdown [19]. On the one hand, the use of technology to join online workshops and for educational and self-development purposes in Qatar is increasing. The government, represented by community centers and the Ministry of Culture and Youth, and other non-governmental organizations, including but not limited to Qatar National Library, *Tomoh* [dream] for Community Development, and Qatar Foundation, played a considerable role in providing virtual activities directed to children, teenagers, and adults. Participants may have been engaged in several local and international virtual programs promoting skills-building, arts and crafts, and participating in online tournaments. On the other hand, using technology for online gaming may not have the same positive effect, even if considered a social interaction through a screen. Several studies found that using technology for online gaming can lead to symptoms of internet gaming disorder post pandemic [20], and may trigger aggressive behaviors, compared to using technology for information search [21].

Natural disasters can cause changes in children and adolescents' daily routines, which may lead to adjustment disorder. According to the Diagnostic Statistical Manual [DSM-5], adjustment disorder presents itself when a stressor may affect a single individual or an entire family or larger community group. Symptoms such as sleep distortion, boredom, wanting to be alone, feeling stressless, and overall electronic usage are developed within three months of a natural disaster such as a pandemic (DSM-5). Studies showed, "Compared to adults, children and adolescents are less well equipped with adaptive coping strategies and ability to control emotions," causing maladjustment [22] (p. 2). However, when the right coping strategy is adopted, children and adolescents reported increase in resilience, causing negative symptomology of maladjustment during natural disasters [22]. Similar to what is reported in this study, most children and adolescents screened for mild adjustment disorder but also managed home isolation and social distancing with activating coping strategies. The causes of higher scoring in adjustment disorder are linked to higher use of electronic devices as it is considered one of the diagnostic criteria. In this study, almost half of participants increased use of electronic devices often or all the time respectively. However, we can identify in this study that participants utilized devices for three primary purposes:

- Reducing boredom through video or online gaming
- Reducing time spent alone by socializing with family or friends
- Maintaining self-development by joining online courses and workshops

We also note studies that found higher use of technology did not evaluate time spent for educational purposes [19]. Thus, it is essential to note that people generally rely on technology for education [19], keeping up to date with news, or staying connected with long-distance relationships. This, in turn, can lower the chances of being disrupted, isolated, and unable to control disaster outcomes. So, technology has different purposes, not all being negative, addictive, or stress-inducing.

In the case of conversing with parents regarding pandemic related information, studies reported that it can help with reducing COVID-19 related stress in children and adolescents [19, 23]. A qualitative study surveyed 210 parents and reported that parents have an essential role in motivating children to adopt personal hygiene behaviors and being a source of coping socialization [23]. The study also underlined the importance of reporting pandemic recommendations to parents to scaffold them into child-appropriate discussions [23]. Similarly, almost 80% of this study's participants reported choosing to receive information often or all the time from parents and official channels instead of rumors from peers. As for its effects on adolescents with a higher risk of anxiety, engaging in conversations with parents can lead to reducing avoidance and distraction coping strategies when dealing with anxiety [24]. One study reported that adolescent-mother communication could endorse positive coping when dealing with emotions [25].

Additionally, participants in this study have reported playing with siblings, parents, or pets as a coping mechanism. We conclude that the outcome of this coping strategy not only has beneficial psychological or physical effects on the individual but can also positively affect others in the immediate social environment [13], that is, the home. Our study found that the majority of participants screened negatively in separation anxiety. As for the effects of playing with pets, a qualitative study in Australia revealed the social, biological, and psychological benefits and challenges of having pets during times of uncertainty and social isolation [14]. While it is challenging to keep up with pet health and training if care is limited during the pandemic, the study reported having pets is "a welcome distraction," "provided encouragement for spending time as a family," and "having a reason to get up" [14]. Similar to this study's findings, pets offer social support and are considered part of coping through play. Taking care of pets can also encourage a sense of responsibility, a reason to show affection, and an active routine for children or adolescents.

Finally, practicing gardening, arts and crafts, and cooking can reduce stress in individuals experiencing trauma or natural disasters. According to Wahl-Alexander and Sinelkikov (2013), "activities that do not focus on verbal recollection of traumatic memories, like art therapy, can be important in the recovery process" and as coping strategies to reduce stress and anxiety (p. 25) [26]. Our study findings indicates the link between having a hoppy and mental health management.

## Implications for practice

The study is unique as it brings the voices of a large number of participants in national research screening for psychological effects during home isolation and social distancing while identifying coping mechanisms adopted by participants and their families. This study recommends several coping strategies for children and adolescents during pandemics and re-adjustment periods. It is important to understand the pivotal role of technology and culture in shaping coping to future generations. For one, results indicated that technology plays a significant role during the COVID-19 pandemic in adopting some positive practices. As for culture, findings document the importance of family cohesion and spirituality, and recommends parents set a routine for family quality shared-time without the presence of pandemic. This can be a protective factor for any future crisis such as endemics and pandemics. Similarly, mastering any type of hobby can lower anxiety and improve tactical decision making. Parents, educators, and practitioners can play an essential role in directing children and adolescents to discover different hobbies, as it helps with emotional management. I.e., collaboration between the educational and healthcare sectors is crucial for healthy childhood in general and in pandemics times. Finally, reporting on the importance of physical exercise, parents and guardians should be modeling and encourage children to adopt exercise in their daily routine to mitigate depressive symptoms and improve their health and longevity.

## Limitations of the study

The study has some limitations. First, researchers cannot control and monitor the ways parents manage the questionnaire. I.e., they cannot be sure of the parent answered on behalf of the child or not. Second, the qualitative component reports on one aspect of coping, which is what did they do. It does not ask more questions to explore the reasons behind these practices. Therefore, we recommend a future study that explores in-depth experiences of children during crisis times.

## Data Availability

The datasets used and/or analyzed during the current study are available from the corresponding author on reasonable request.

## Footnotes

1 For more information on the full report, please see: Zainel, A. A., Qotba, H., Al-Maadeed, A., Al-Kohji, S., Al Mujalli, H., Ali, A., … & Al-Baghdadi, T. (2021). Psychological and coping strategies related to home isolation and social distancing in children and adolescents during the COVID-19 pandemic: Cross-sectional study. JMIR Formative Research, 5(4), e24760.

2 Early morning prayer.

3 *Al-Baqarah*, is the second and longest chapter of the Holy Quran.
